# All-Inorganic p−n Heterojunction Solar Cells by Solution Combustion Synthesis Using N-type FeMnO_3_ Perovskite Photoactive Layer

**DOI:** 10.3389/fchem.2021.754487

**Published:** 2021-09-29

**Authors:** Ioannis T. Papadas, Apostolos Ioakeimidis, Ioannis Vamvasakis, Polyvios Eleftheriou, Gerasimos S. Armatas, Stelios A. Choulis

**Affiliations:** ^1^ Molecular Electronics and Photonics Research Unit, Department of Mechanical Engineering and Materials Science and Engineering, Cyprus University of Technology, Limassol, Cyprus; ^2^ Department of Public and Community Health, School of Public Health, University of West Attica, Athens, Greece; ^3^ Department of Materials Science and Technology, University of Crete, Heraklion, Greece

**Keywords:** inorganic perovskites, solution combustion synthesis, FeMnO3, NiO, p-n junction, functional metal oxides, inorganic solar cells, photoactive nanomaterials

## Abstract

This study outlines the synthesis and physicochemical characteristics of a solution-processable iron manganite (FeMnO_3_) nanoparticles via a chemical combustion method using tartaric acid as a fuel whilst demonstrating the performance of this material as a n-type photoactive layer in all-oxide solar cells. It is shown that the solution combustion synthesis (SCS) method enables the formation of pure crystal phase FeMnO_3_ with controllable particle size. XRD pattern and morphology images from TEM confirm the purity of FeMnO_3_ phase and the relatively small crystallite size (∼13 nm), firstly reported in the literature. Moreover, to assemble a network of connected FeMnO_3_ nanoparticles, *β*-alanine was used as a capping agent and dimethylformamide (DMF) as a polar aprotic solvent for the colloidal dispersion of FeMnO_3_ NPs. This procedure yields a ∼500 nm thick FeMnO_3_ n-type photoactive layer. The proposed method is crucial to obtain functional solution processed NiO/FeMnO_3_ heterojunction inorganic photovoltaics. Photovoltaic performance and solar cell device limitations of the NiO/FeMnO_3_-based heterojunction solar cells are presented.

## Introduction

A major source of renewable energy is solar energy (Ellabban et al., 2014). Nevertheless, the production of fuels and electricity from solar power is still costly, mainly because of the materials used in building the cells (Ellabban et al., 2014; Hussein, 2015). Except for Si and copper indium gallium selenide solar cells (CIGSSe), which are principal targets for photovoltaic (PV) applications, CdTe and GaAs are also significant photoactive materials. However, the mass production of such photovoltaics is limited due to the high production costs, the indirect bandgap energy (for Si) and the dependence on elements which are expensive (In, Ga, Te) or even hazardous (As, Cd) (Rohatgi, 1996; Mitzi et al., 2011; Kosten et al., 2013; Wang, 2014). Contrarily, hybrid lead halide perovskite solar cells (PVSCs) have been in the epicenter of the current solar cell research because of their facile fabrication process and due to the fact that they appear a high PCE of over 23% (Jung et al., 2019). However, PVSCs have disadvantages as well, that is, ultraviolet light absorption, and humidity and atmospheric oxygen affecting decomposition (Li and Liu, 2017; Fu et al., 2018387). Along with the presence of toxic lead, these factors restrain the advancement of the PVSCs. Therefore, it is essential to find alternatives for inorganic PVs that use inexpensive, eco-friendly and Earth abundant photoactive materials with appropriate semiconductor structure, while at the same time seek improvements in solar cell efficiency.

Metal oxide (MeOx) based solar cells have the potential to resolve some of the issues which arise in conventional solar cells. The all-oxide perspective is advantageous due to its excellent chemical stability, minor toxicity and ample quantities of metal oxides that effectively permit the manufacturing of solar cells under ambient conditions (Pérez-Tomás et al., 2018). MeOx are typically used as functional layers in solar cells such as transparent conducting front electrodes (ITO, FTO), (Kim et al., 1999; Han et al., 2007) electron (TiO_2_, SnO_2_, ZnO, Fe_2_O_3_ etc.) (Seo et al., 2018; Xiong et al., 2018; Shin et al., 2019; Papadas et al., 2019) or hole (Cu:NiOx, CuGaO_2_, NiCo_2_O_4_, CuLi:NiCo_2_O_4_ etc.) transporting layers, (Galatopoulos et al., 2017; Papadas et al., 2018a; Papadas et al., 2018b; Ioakeimidis et al., 2019) whereas a very small number of MeOx have been used as photoactive layers, primarily Cu_2_O, CuO and Co_2_O_3_ (Pérez-Tomás et al., 2018).

Ferroic semiconductors are now being continually included in the list of materials which are employed to investigate and push the efficiency limits in all-oxide photovoltaics. Ferroic semiconductor materials are considered to be light absorbers, such as Pb(Zr,Ti)O_3_ (energy band gap (E_g_) = 3.63 eV) (Pérez-Tomas et al., 2019), KNbO_3_ (E_g_ = 3.8 eV) (Grinberg et al., 2013) and BiFeO_3_ (E_g_ = 2.67 eV) (Papadas et al., 2015a). However, what seems to confine their implication to solar cells is their wide energy bandgap that results to low absorption of visible light and, thus, low conductivity. Other ferroelectric oxide semiconductors, like Bi_2_FeCrO_6_ (Nechache et al., 2014) and BiMnO_3_ (Sun et al., 2017), have a suitable bandgap between 1 and 2 eV and are considered as more efficient in absorbing solar light. It is worth mentioning that a power conversion efficiency (PCE) up to ∼8.1% has been recently achieved using a single ferroelectric Bi_2_FeCrO_6_ layer fabricated by pulse laser deposited technique with the following structure: SrTiO_3_/SrRuO_3_/Bi_2_CrFeO_6_/ITO (Nechache et al., 2014). However, without the mentioned method of the thin film deposition which is considered to be a very complicated and energy demanding procedure, it is very unlikely to gain high PCE values (Calnan, 2014). Furthermore, the usage of the low-bandgap KBiFe_2_O_5_ material (E_g_ ∼1.6 eV) in the photovoltaic cells appears to be restricted due to low PCE (∼3 10^–3^%) (Zhang et al., 2013). Therefore, the above mentioned examples demonstrate that further research is needed in finding more efficient narrow-bandgap, non-toxic and low-cost materials for solar cell devices.

The sol-gel synthesis has been the most frequently used method in the manufacture of MeOx. Nevertheless, to achieve crystallinity and to guarantee an efficient charge–carrier mobility, for metal oxide based active layers, high temperatures are necessary, which increases the cost of manufacturing and also restricts printable applications. These restrictions call for alternative techniques which could operate at lower temperatures. Compared to sol-gel synthesis of MeOx nanoparticles (NPs), the solution combustion synthesis (SCS) of NPs displays considerable advantages, such as use of a simple experimental setup, production of NPs with high crystallinity and pure phase, and exact control of the size and crystal structure of the particles by simple adjusting the fabrication conditions. (Suresh et al., 1991; Mimani and Patil, 2001; Patil et al., 2002a; Bansal, 2005; Deganello et al., 2009; Jadhav et al., 2011; Jiang et al., 2014) The SCS seems to be adaptable and effective for the growth of high crystalline MeOx layers at relative lower temperatures. As an exothermic procedure with a high rate of heat release, the necessity for high temperatures is circumvented and high purity MeOx NPs can be produced at moderate reaction conditions. In SCS process, the metal salts (e.g., nitrates) dissolved in saturated aqueous or alcoholic solutions act as oxidizing agents and react with organic fuels (such as urea, glycine, acetylacetonate, citric acid etc.) under relatively lower temperatures compared to other commonly used solution process methods to give rise to a combustion reaction and to produce the corresponding metal oxide NPs (Kim et al., 2011; Hsieh, 2014; Yu, 2015).

Iron manganite (FeMnO_3_, FMO) is a mixed perovskite material with the chemical formula ABO_3_, where the Fe atom is placed at the center of a cube formed by eight corner-sharing MnO_6_ octahedra (Habibi and Mosavi, 2017). FeMnO_3_ has been examined for applications such as lithium-ion batteries, catalysis, humidity sensors, energy storage and antibacterial devices (Doroftei et al., 2014; Cao et al., 2016; Cetin et al., 2019; Vasiljevic et al., 2020a; Nikolic et al., 2020). A large number of synthesis methods, such as co-precipitation, hydrothermal, ball milling, solid state reaction and sol-gel chemistry, have all been employed for the fabrication of FeMnO_3_ materials (Sundari et al., 2013; Doroftei et al., 2014; Cao et al., 2016; Soni and Pal, 2016; Bin et al., 2017; Mungse et al., 2017; Gowreesan and Ruban Kumar, 2017; Saravanakumar et al., 2018; Cetin et al., 2019; Fix, 2019; Lobo and Rubankumar, 2019; Vasiljevic et al., 2020a; Nikolic et al., 2020; Vasiljevic et al., 2020b). Despite this, not all these techniques are viable to synthesize FeMnO_3_ nanomaterials, as there are some drawbacks such as the expense of the source materials, chemical non-uniformity, high impurity, aggregated nanoparticles, and non-stoichiometry of some ferrite systems (Buonsanti et al., 2012; Alves et al., 2013; Bennet et al., 2016; Skliri, 2018). FeMnO_3_ is a semiconductor which consists of plentiful and environmentally friendly elements with an ideal direct optical bandgap (∼1.5 eV) to absorb solar photons, while it has a deep lying valence band (VB ∼ 5.3 eV) (Skliri, 2018) that corresponds well to the VB edges of several p-type materials (e.g., CuO, NiCo_2_O_4_ etc.) (Savva et al., 2017; Papadas et al., 2018b). Additionally, it has high photochemical stability which is necessary for long-term optoelectronic devices while furthermore its intrinsic electric polarization field can enable charge-carrier separation within the semiconducting structure. Such characteristics make FeMnO_3_ a promising light absorber for optoelectronic uses. On the other hand, between the numerous metal oxides that have been used as p-type active layers, nickel oxide (NiO) is a promising candidate for PVs due to its excellent electrochemical behavior (Kerli and Alver, 2016). NiO demonstrates a rock salt structure and exhibits adequate p-type conductivity with a wide bandgap in the range of 3.5 eV (Chrissanthopoulos et al., 2011; Mahmood et al., 2011; Zhang, 2015). NiO has been reported as p-type material in all-oxide solar cells in combination with the n-type TiO_2_ and ZnO materials, while also solution combustion synthesized NiOx is commonly used as HTL in perovskite solar cells (Warasawa et al., 2013; Kawade et al., 2015; Kerli and Alver, 2016; Karsthof et al., 2016; Galatopoulos et al., 2017; Patel, 2017; Ukoba et al., 2018).

In this work, SCS of FeMnO_3_ NPs is presented while indicating that tartaric acid can be used as a fuel and nitrate as an oxidizer agent. FeMnO_3_ NPs characterized by an average size of ∼13 nm and a narrow particle-size distribution, were prepared using a low cost SCS process (6 h calcination at 450°C in air). The as-synthesized FeMnO_3_ NPs were then functionalized with *β*-alanine and the ligand-capped NPs enabled the formation of compact and functional layers. These films were used, for the first time, as n-type photoactive materials and were incorporated in p-n heterojunction of all-oxide solar cells. For the purposes of this study, nanostructured NiO films were also synthesized by SCS method and applied as a p-type layer in the following structure ITO/NiO/FeMnO_3_/Cu. The corresponding MeOx PVs show a high V_oc_ of 1.31 V with adequate FF of 54.3% and limited short current of 0.07 mA cm^−2^ resulting to a PCE of 0.05%. Electrical characterizations by impedance spectroscopy reveled a high charge recombination resistance inducing high V_oc_, whereas the limited current density is ascribed to the high charge transport resistance. Despite the low PCE values, these results provide a framework for further optoelectronic properties research on eco-friendly and cost-effective photoactive layers for fabrication of all solution processable inorganic photovoltaics.

## Experimental

*Materials:* Pre-patterned glass-ITO substrates (sheet resistance 4 Ω/sq) were purchased from Psiotec Ltd. All the other chemicals used in this study were purchased from Sigma Aldrich.

*Solution combustion synthesis (SCS) of FeMn O*_*3*_ *NPs:* For the synthesis of FeMnO_3_ NPs, 0.5 mmol Mn(NO_3_)_2_.4H_2_O, 0.5 mmol Fe(NO_3_)_3_.9H_2_O and tartaric acid were blended in 5 ml of 2-methoxy ethanol solution. Subsequently, 150 μl HNO_3_ (69% wt HNO_3_) were added slowly into the mixture, and the solution stirred up to almost complete homogeneity. The whole solution was left under stirring for at least 3 h at room temperature (RT). The molar ratio of the total metal nitrates and tartaric acid was 1. Thereafter, the precursor solution was heated at 100°C under consecutive stirring until complete evaporation of the solvent. The dry black powder was then used for the combustion synthesis of the FeMnO_3_ NPs in ambient atmosphere at 450°C in a preheated oven for 6 h, so that the combustion process be completed and then left to cool down at room temperature.

*Perovskite FeMn O*_*3*_ *films preparation:* The prepared FeMnO_3_ (FMO) NP powder was used for the preparation of FMO dispersion for the deposition of corresponding film by spin coating technique. Firstly, the surface of NPs was modified with *β*-alanine. Briefly, as-made FMO NPs (50 mg) were added in 4 ml of deionized (DI) water containing *β*-alanine (10 mg), and the pH of the solution was adjusted to 4.2 with 1M HNO_3_. (Papadas et al., 2015b; Skliri, 2018) To secure that NPs will transfer to the liquid phase and form a stable suspension, typically within 1 day, the resulting mixture was then intensively stirred at RT. The dispersion was aided with probe sonicator for about 30 min. To a stable colloidal dispersion of 30 mg ml^−1^ be formed, the alanine-capped FMO NPs were isolated by centrifugation, rinsed several times with DI water, and finally dispersed in DMF. The obtained homogenous dispersion was then drop-casted and subsequently was spin coated on the top of NiO layer at 3,000 rpm for 40 s. The process of FeMnO_3_ film formation was repeated about ten times to obtain a desired thickness of about 500 nm. To assemble a network of tightly connected metal oxide NPs, the deposition of the FeMnO_3_ films was accomplished by spin coating technique of the colloidal NPs, followed by thermal annealing at 300°C for 30 min. In this way, *β*-alanine can enable direct NP–NP interactions upon ligand removal at growth temperature due to its small size, thus yielding high strength films consisted of firmly interconnected NP networks. This strategy is very important to obtain functional metal oxides photovoltaic devices with good charge transfer properties.

*NiO NPs synthesis and films preparation by SCS:* For the solution combustion synthesis of NiO, 1 mmol of Ni(NO_3_)_2_·6H_2_O were dissolved in 2.5 ml of 2-methoxyethanol. After the solution was stirred at 50°C for 1 h, 0.1 mmol of acetylacetone was added to the solution, and the whole solution was allowed under further stirring for 1 h at RT. Spin coating technique was applied for the fabrication of the precursor films on the various substrates. The precursor’s solution was spin coated at 3,000 rpm for 40 s. The resulting light green colored films were dried at 100°C for 5 min and used as a precursor for the combustion synthesis of NiO NPs. Subsequently the obtained films were heated at 300°C in ambient atmosphere for 1 h in a preheated hot plate to complete the combustion process and then left to cool down at room temperature, forming a ∼50 nm thin layer.

*Device fabrication:* The metal oxides solar cells under study were ITO/NiO-NPs/FeMnO_3_-NPs/Cu. ITO substrates were sonicated in acetone and subsequently in isopropanol for 10 min and then heated at 100°C on a hot plate for 10 min before use. The substrates were further treated with ozone for 10 min to achieve a better contact with the active layer by reducing the contact resistance. To fabricate the devices, a layer of NiO as p-type and FeMnO_3_ as n-type side of the p-n junction were formed in sequence. The deposition of corresponding metal oxides films was described in detail above. Finally, 200 nm Cu layers were thermally evaporated through a shadow mask to finalize the devices, giving an active area of 0.9 mm^2^.

*Characterization:* Thermogravimetric Analysis (TGA) were performed on a Shimadzu Simultaneous DTA-TG system (DTG-60H). Thermal analysis was conducted from 40 to 600°C in air atmosphere using air gas with a flow rate of 200 ml min^−1^ and a heating rate of 10°C min^−1^. X-ray diffraction (XRD) patterns were collected on a PANanalytical X´pert Pro MPD powder diffractometer (40 kV, 45 mA) using Cu Kα radiation (λ = 1.5418 Å). Transmission electron microscope (TEM) images and electron diffraction patterns were recorded on a JEOL JEM-2100 microscope with an acceleration voltage of 200 kV. The samples were first gently ground, suspended in ethanol, and then picked up on a carbon-coated Cu grid. Quantitative microprobe analyses were performed on a JEOL JSM-6390LV scanning electron microscope (SEM) equipped with an Oxford INCA PentaFET-x3 energy dispersive X-ray spectroscopy (EDS) detector. Data acquisition was performed with an accelerating voltage of 20 kV and 60 s accumulation time. Absorption measurements were performed with a Schimadzu UV-2700 UV-Vis spectrophotometer. For UV-VIS and PL measurements, thick films of FeMnO_3_ NPs have been fabricated on top of the quartz substrates employing the spin coating method. UV–vis/near-IR diffuse reflectance spectra were recorded with a Schimadzu UV-2700 UV-Vis spectrophotometer, using BaSO_4_ powder as a 100% reflectance standard. The energy bandgap (E_g_) of the samples were estimated from Tauc plots of (Fhv)^2^ as a function of photon energy (hv), where F is the Kubelka–Munk function of the reflectance (R): F=(1−R)^2^/(2R) (Kubelka, 1948). The thickness of the films were measured with a Veeco Dektak 150 profilometer. The PL measurements were performed on FeMnO_3_ film on quartz substrate at an excitation wavelength of 400 nm. Photoluminescence (PL) spectrum was obtained at room temperature on a Jobin-Yvon Horiba FluoroMax-P (SPEX) spectrofluorimeter (Singapore) equipped with a 150 W Xenon lamp and operated from 300 to 900 nm. The current density-voltage (J-V) characteristics were characterized with a Botest LIV Functionality Test System. Forward bias scans were measured with 10 mV voltage steps and 40 msec of delay time. For illumination, a calibrated Newport Solar simulator equipped with a Xe lamp was used, providing an AM1.5G spectrum at 100 mW/cm^2^ as measured by a certified oriel 91150 V calibration cell. A shadow mask was attached to each device prior to measurements to accurately define the corresponding device area. EQE measurements were performed by Newport System, Model 70356_70316NS. Atomic force microscopy (AFM) images were obtained using a Nanosurf easy scan two controller under the tapping mode. Electrochemical Impedance Spectroscopy (EIS) and Mott-Schottky measurements were performed using a Metrohm Autolab PGSTAT 302N, where for the EIS a red light-emitting diode (LED) (at 625 nm) was used as the light source calibrated to 100 mW/cm^2^. For EIS a small AC perturbation of 20 mV was applied to the devices, and the different current output was measured throughout a frequency range of 1 MHz-1 Hz. The steady state DC bias was kept at 0 V throughout the EIS experiments. Mott-Schottky measurements on FeMnO_3_ films were performed in a 0.5 M Na_2_SO_4_ aqueous electrolyte (pH = 7) using a Metrohm Autolab PGSTAT 302N potentiostat. A three-electrode set-up, with a platinum plate (1.0 × 2.0 cm^2^) and a silver-silver chloride (Ag/AgCl, 3M KCl) as the counter and reference electrodes, respectively, was adopted to study the samples. The capacitance of the semiconductor/electrolyte interface was obtained at 1 kHz, with 10 mV AC voltage perturbation. All the experiments were conducted under dark conditions. The measured potential vs the Ag/AgCl reference electrode was converted to the normal hydrogen electrode (NHE) scale using the formula: E_NHE_ = E_Ag/AgCl_ + 0.210 V. The working electrode for impedance-potential measurement was fabricated as follows, 10 mg of FeMnO_3_ NPs was dispersed in 1 ml DI water and the mixture was subjected to sonication in a water bath until a uniform suspension was formed. After that, 100 µl of the suspension was drop-casted onto the surface of fluorine-doped tin oxide (FTO, 9 Ω/sq) substrate, which was masked with an epoxy resin to expose an effective area of 1.0 × 1.0 cm^2^. The sample was dried in a 60°C oven for 30 min.

## Results and Discussion

The solution processing technique provides an extensible low cost deposition procedure to fabricate high quality metal-oxide films and to replace costly and time consuming vacuum-deposition methods (Cochran et al., 2019). SCS has recently been employed in the low temperature manufacture of spinel nickel cobaltite (NiCo_2_O_4_) thin films as hole transport layers (HTLs) in inverted p-i-n perovskite solar cells (Papadas et al., 2018b). SCS possesses the benefit of speedily creating homogenous metal oxide materials of fine grain size. Most notably however, at a lower temperature than standard solid–state reaction, sol-gel and co-precipitation techniques (Chick et al., 1990; Rajeshwar and de Tacconi, 1998). Metal nitrates are distinguished for their capacity to synthesize metal oxides films of superior quality. Furthermore, it is important to choose the appropriate fuel agent for combustion, so as to circumvent the creation of sizeable clusters and particle agglomeration (Bansal, 2005; Verma et al., 2008). Tartaric acid was therefore used as the fuel agent in this work as it results in the formation of a single-crystalline phase of FeMnO_3_. In general, tartaric acid leads to the formation of stable heterometallic polynuclear complexes (Selbach et al., 2007) because of its carboxylate and hydroxyl groups which can bind different metal ions from the solution, such as Mn^2+^ and Fe^3+^ (Wang et al., 2011). Basically, the growth of FeMnO_3_ NPs is the result of combustion reaction of these polynuclear complexes while being heated in the presence of concentrated HNO_3_ (Patil et al., 2002b; Cochran et al., 2019).

### Synthesis and Characterization FeMnO_3_ Nanoparticles

**FIGURE 1:**
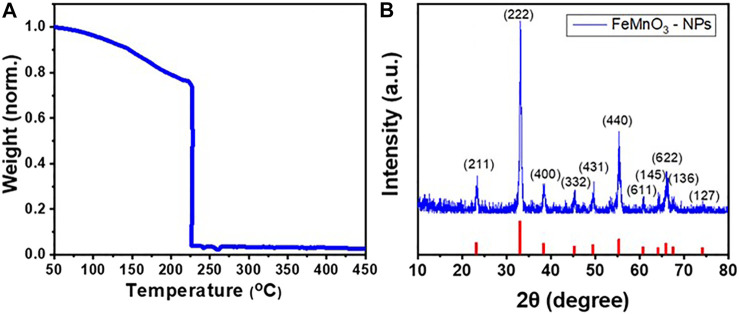
**(A)** TGA profile of the as-prepared FeMnO_3_ material synthesized via solution combustion process. The weight loss was normalized to the initial sample mass. **(B)** XRD pattern of FeMnO_3_ NPs obtained at 450°C annealing temperature.

To achieve the solution combustion synthesis of FeMnO_3_ NPs, tartaric acid and metal nitrates precursors were dissolved in 2-methoxyethanol. The precursor’s solution was heated at 100°C under stirring until complete evaporation of the solvent. The obtained gel product was then analyzed by thermogravimetric analysis (TGA). The thermal behavior of the Mn/Fe-tartaric compound was observed by TGA, employing a heating rate of 10°C min^−1^ in ambient air. As illustrated in Figure 1A, the reaction shows an acute sudden mass loss at ∼230°C, noted in TGA curve, which is associated with a strong exothermic release of energy during the combustion process. In our study, the as-synthesized material was crystallized well to the perovskite phase in ambient atmosphere at 450°C in a preheated oven for 6 h.

The XRD measurement confirmed the crystallinity and phase purity of the FeMnO_3_ NPs produced through the SCS method. Figure 1B depicts the XRD pattern of nanocrystalline FeMnO_3_ obtained at 450°C soaking temperature. All the diffraction peaks compare well with the reported cubic iron manganite structure (JCPDS card #75–0,894) with a = b = c = 9.4 Å and α= β = γ = 90° unit cell parameters. No peaks from impurity phases, like MnO or Fe_2_O_3_, were observed in XRD pattern, showing the phase purity of the sample. The mean FeMnO_3_ crystallite size is estimated at ∼15 nm using the Scherrer`s equation and peak broadening of the (222) reflection.

**FIGURE 2:**
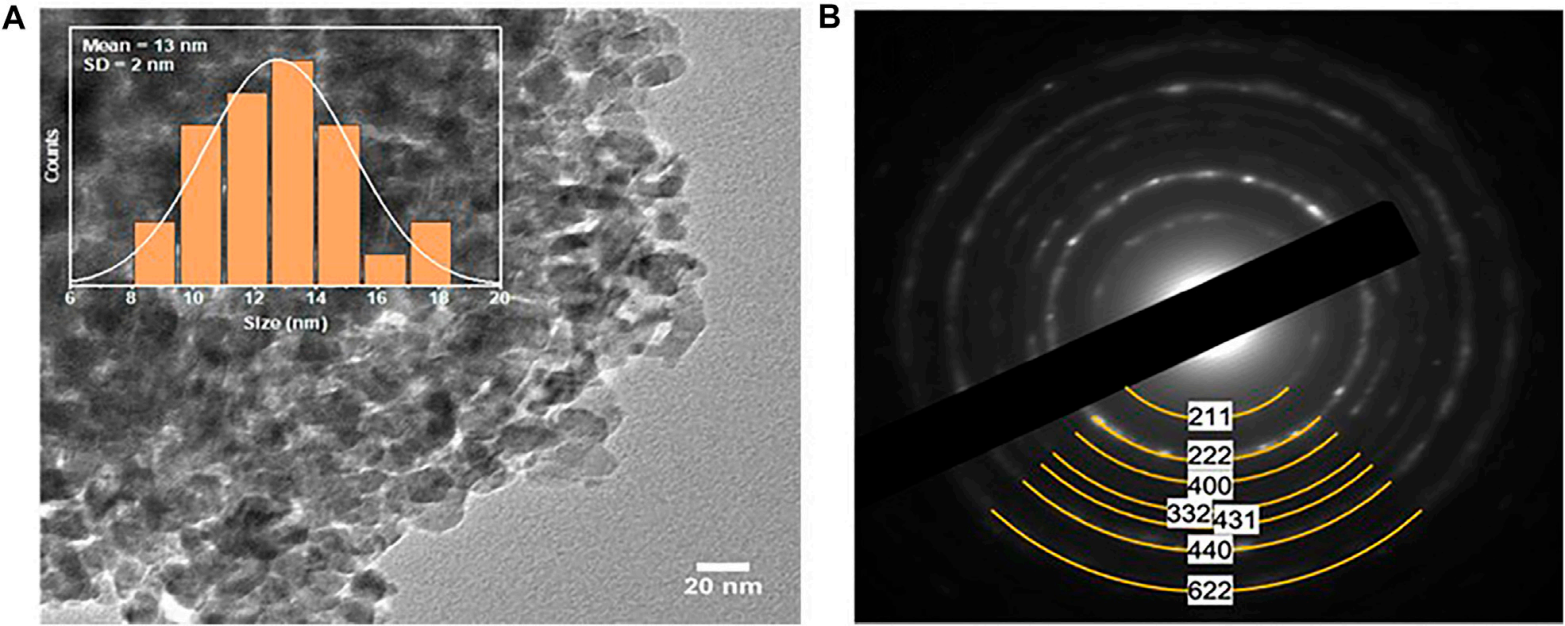
**(A)** Representative TEM image (inset size distribution histogram of the FeMnO_3_ NPs, showing an average diameter of 13 ± 2 nm), and **(B)** SAED pattern of the as-synthesized FeMnO_3_ NPs obtained at 450°C.

TEM corroborated the phase purity of the obtained FeMnO_3_ NPs. Figure 2A illustrates a characteristic TEM image of the FeMnO_3_ sample fabricated at 450°C. It depicts that the obtained FeMnO_3_ consists of tightly connected NPs with an average diameter of 13 ± 2 nm in average (inset Figure 2A), which match well to the crystallite size calculated from XRD. The crystal structure of the FeMnO_3_ was then examined by selected-area electron diffraction (SAED). The SAED pattern recorded from a small area of the FeMnO_3_ sample (Figure 2B) indicates a series of broad concentric diffraction rings, which can be assigned to the cubic phase of FeMnO_3_ (Li et al., 2014). In line with XRD results, no other crystal phases were detected by means of electron diffraction. Furthermore, characterization of the composition of FeMnO_3_ with EDS analysis revealed a Fe:Mn atomic ratio close to 1:1, in agreement with the stoichiometry of FeMnO_3_ compound (Supplementary Figure S1).

**FIGURE 3:**
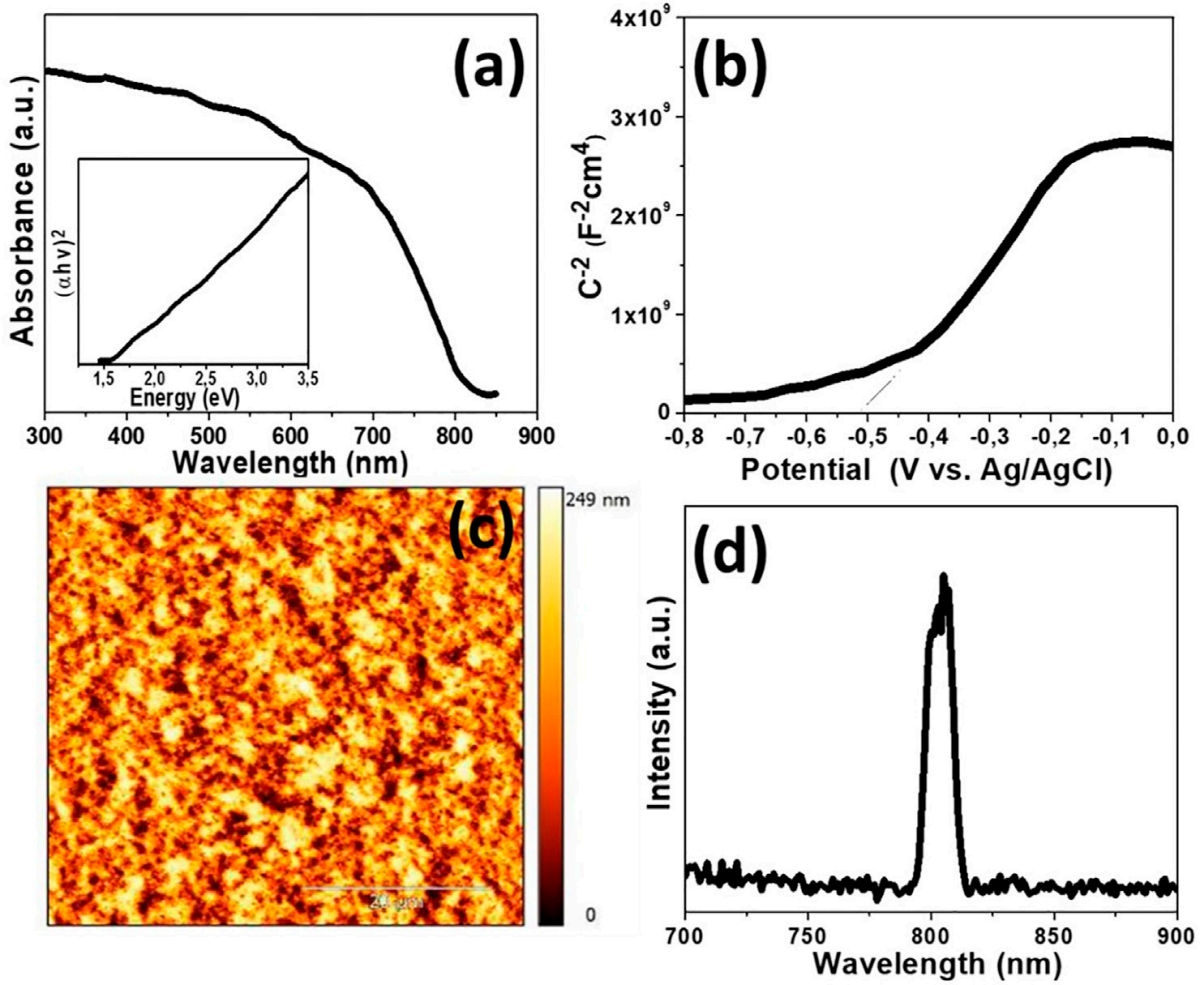
**(A)** UV-Vis/NIR absorption spectrum of FeMnO_3_ NPs [Inset: the (Fhv)^2^ versus hv plot derived from optical absorption spectra]. **(B)** Mott-Schottky plot of the inverse square space-charge capacitance (1/C_SC_^2^) as a function of applied voltage (E) relative to the redox potential of Ag/AgCl (3 M KCl) for the FeMnO_3_ NPs. **(C)** Typical AFM image of the ITO/FeMnO_3_ film after SCS synthesis at 450°C (The scale bar is 20 μm). **(D)** Room-temperature PL emission spectra of the FeMnO_3_ NPs.

The electronic structure of as-prepared FeMnO_3_ was also examined by diffuse reflectance ultraviolet-visible/near-IR (UV-vis/NIR) spectroscopy. Figure 3A shows the UV-vis/NIR absorption spectrum for FeMnO_3_ NPs synthesized at 450°C by SCS. This sample shows an acute optical absorption onset in the near IR region (∼805 nm), which is associated with an energy gap at ∼1.54 eV, as determined by Tauc`s plots [(Fhv)^1/2^ versus photon energy (hv), where F, h, and v are the Kubelka-Munk function of the reflectance, Plank constant and light frequency, respectively] (Kubelka, 1948), see inset of Figure 3A.

**FIGURE 4:**
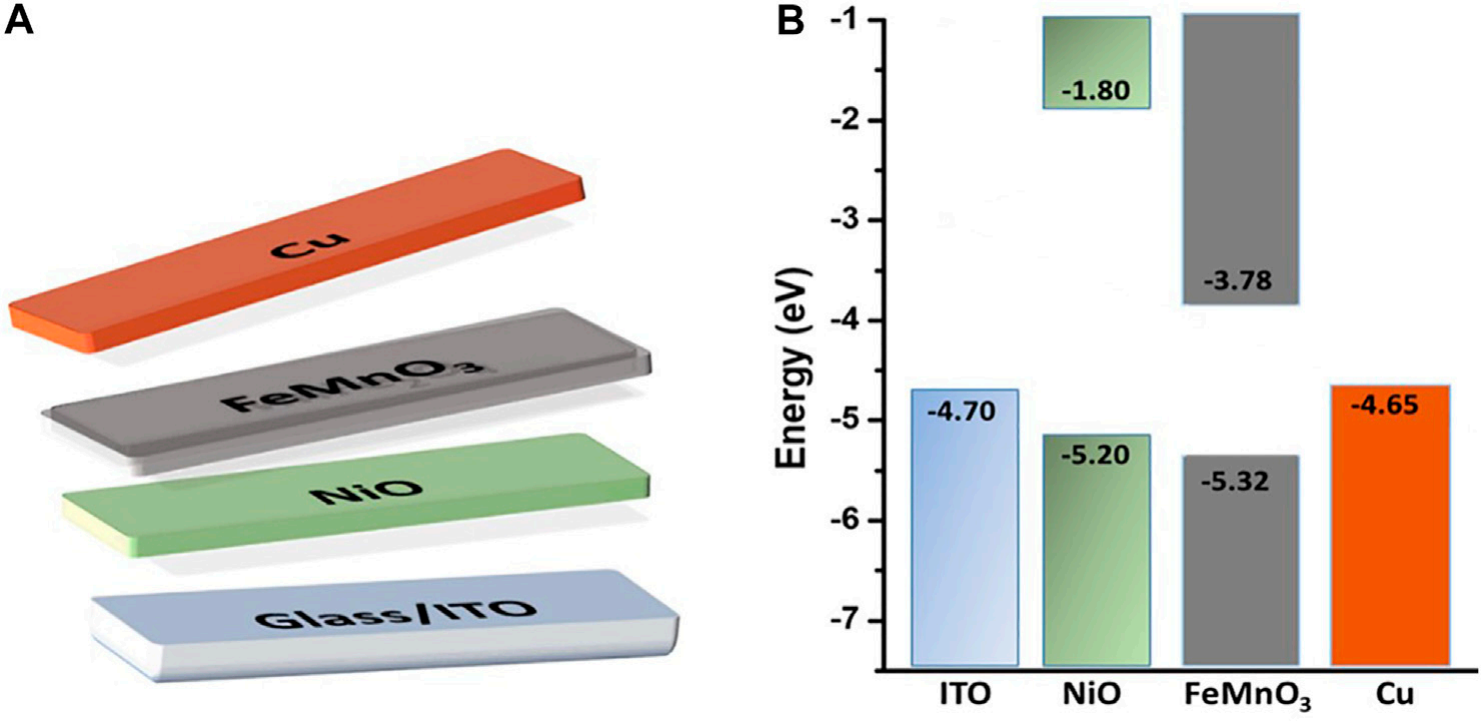
**(A)** Schematic representation, and **(B)** the corresponding energy level diagram of each component of the ITO/NiO/FeMnO_3_/Cu device.

Electrochemical impedance spectroscopy (EIS) thereafter was employed to examine the position of the conduction band (CB) and valence band (VB) edges of FeMnO_3_ material. Figure 3B shows the ensuing Mott-Schottky plot and the matching fit of the linear part of the inverse square space-charge capacitance (1/C_sc_^2^) as a function of potential (E). The FeMnO_3_ reveals a positive linear slope, showing n-type conductivity, where electrons are majority carriers. By using extrapolation to 1/C_SC_^2^ = 0, the flat-band potential (E_FB_) of FeMnO_3_ NPs was estimated to be –0.31 eV vs. NHE (pH = 7). Based on the E_FB_ and optical bandgap (as obtained from UV-vis/NIR reflectance data, Figure 3A) values, the energy band edges for FeMnO_3_ NPs are CB: 3.78 eV and VB: 5.32 eV vs. vacuum (Skliri, 2018). This is further highlighted in the energy level diagram shown in Figure 4B, which is based on EIS measured values for FeMnO_3_ and literature data for ITO, NiO and Cu components (Haque et al., 2017). For heavily n-typed doped semiconductors, it can be supposed that the E_FB_ level is very close to the CB edge. Generally, for several n-type semiconductors the CB edge is approximately 0.1–0.3 eV higher than the E_FB_ potential. Therefore, the position of the VB edge was estimated from E_FB_–E_g_. In iron manganite materials there are a number of reports which connect the electron hopping between Fe^+3^-Fe^+2^ and hole hopping between Mn^+2^-Mn^+3^ ions with n-type and p-type of conductivities (Veena Gopalan et al., 2008; Batoo et al., 2009). The findings of these studies suggest that both n-type and p-type charge carriers are anticipated to contribute to the conduction mechanism in FeMnO_3_ structure (Rezlescu and Rezlescu, 1974; Akhtar and Younas, 2012) In our study, the positive slope of the Mott-Schottky plots (Figure 3B) clearly shows that the perovskite iron manganite have n-type behavior (Skliri, 2018).

The smoothness of deposited FeMnO_3_ film plays a crucial role for the formation of a deep depletion region, which is highly desirable for high performing devices. To achieve this β-alanine was used as surface capping ligand for FeMnO_3_ NPs. During this process, the carboxyl (–CO_2_H) groups of β-alanine adjust to the nanoparticle’s surface, whereas the amine (–NH_2_) functional groups inhibit the nanoparticles aggregation whilst stabilizing the colloidal solution. This resulted in the formation of a ∼500 nm thick compact film (Supplementary Figure S2) consisting of a continuous network of tightly interconnected NPs (Supplementary Figure S3) with a relative low roughness of ∼27 nm, as calculated by AFM topography measurements (Figure 3C).

For PL measurements of perovskite material, thick layers of FeMnO_3_ NPs were manufactured on top of the quartz substrates utilizing the spin coating method (for details, see the experimental section). PL spectroscopy is an essential tool for finding the purity and crystalline quality of semiconductors. The PL spectrum of FeMnO_3_ NPs, in Figure 3D, shows an intense near band edge emission at ∼805 nm. This emission peak corresponds to the CB-VB inter-band transition and no additional peaks due to the radiative relaxations from defect sites or impurities were observed in PL spectrum of FeMnO_3_ (Vasiljevic et al., 2020a).

### Photovoltaic Device Characterization

As a proof of concept, the newly developed FeMnO_3_ NP aggregates were used as a n-type photoactive material in a p-n full metal oxides solar cell, with the structure ITO/NiO/FMO/Cu (Figure 4A). Both NiO and FeMnO_3_ materials were synthesized by the solution combustion method, as it is described in the experimental sections, rendering the fabrication process of such solar cells remarkably facile. Lastly, a 200-nm-thick Cu layer was thermally placed on the surface of FeMnO_3_ to complete the device (see Figure 4A).

**FIGURE 5:**
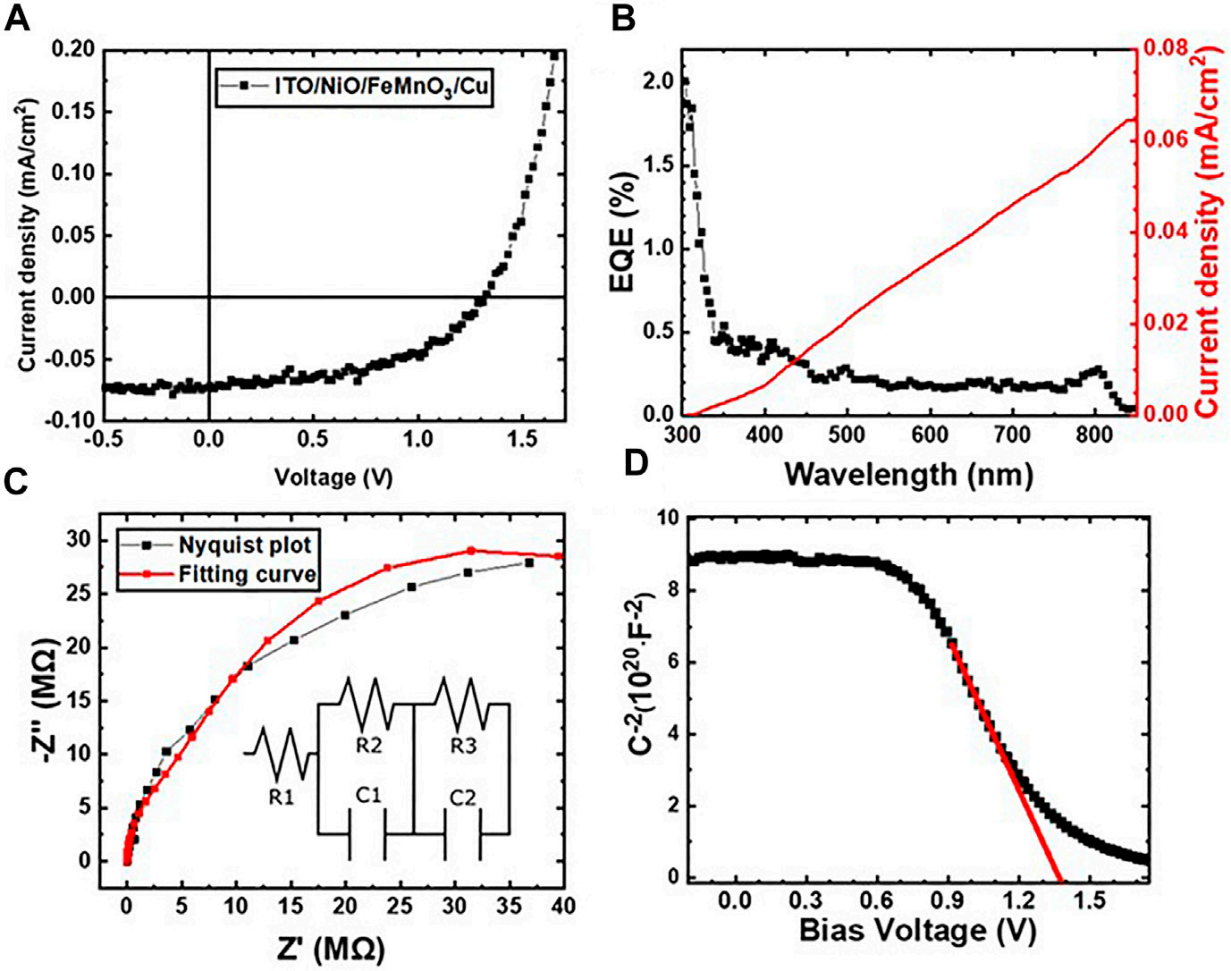
**(A)** J-V plot under illumination conditions for the representative p-n device under this study ITO/NiO/FeMnO_3_/Cu. **(B)** EQE spectrum of NiO−FeMnO_3_ (p–n) heterojunction sandwiched between ITO and Cu electrodes. The right axis represents the integrated photocurrent density of the corresponding device. **(C)** Nyquist, and **(D)** Mott–Schottky plots for the ITO/NiO/FeMnO_3_/Cu device.

Figure 5A shows the J–V curve of the ITO/NiO/FeMnO_3_/Cu device under 1 Sun simulated light (100 mW cm^−2^) where the curve shape evidence the Schottky barrier formation at the junctions. The extracted photovoltaic parameters, open-circuit voltage (V_oc_), short-circuit current (I_sc_), fill factor (FF), and power conversion efficiency (PCE) are listed in Table 1. The device yields a high V_oc_ of 1.31 V with adequate FF of 54.3%, but the generated current density is low (J_sc_ = 0.07 mA cm^−2^) delivering a PCE of 0.05%.

**TABLE 1:** Extracted solar cell parameters from the J–V characterization of the ITO/NiO/FeMnO_3_/Cu device.

Solar cell	V_oc_ (V)	J_sc_ (mA/cm^2^)	FF (%)	PCE (%)
ITO/NiO/FMO/Cu	1.31	0.07	54.3	0.05

Among the studied all-oxide ferroic solar cells, that were fabricated by the same solution processes, the highest PCE was obtained by FeMnO_3_-based solar cell (∼0.05%), which is notably higher than those of the NiO/BiFeO_3_ (∼0.025%) (Chatterjee et al., 2014) and pure Pb(ZrTi)O_3_ (∼0.00008%) based solar cells (Paik et al., 2016). Furthermore, the obtained V_oc_ of the FeMnO_3_-based solar cell (∼1.31 V) is also much higher with respect to the solar cells using BiFeO_3_ (∼0.41 V) (Chatterjee et al., 2014) and Pb(ZrTi)O_3_ (∼0.6 V) as a light absorber, respectively (Paik et al., 2016). Consequently, we suggest that FeMnO_3_ could be a potential candidate for solar cell applications.

To examine the spectral response of the device, external quantum efficiency (EQE) measurements were conducted, while the results being presented in Figure 5B along with the respective integrated photocurrent response. The spectral response of EQE reveals that the photo-generated current is produced in both NiO and FeMnO_3_ layers with the spectrum correspondingly match the optical absorption spectra of the respective NiO and FeMnO_3_ films. Specifically, we observe photocurrent generation onsets in the ultraviolent (300–350 nm) and near IR (800–850 nm) regions, which correspond to the acute optical absorption onset of NiO (∼355 nm) and FeMnO_3_ (∼805 nm), respectively (Tang et al., 2018). Thus, in the device structure under investigation the p-type NiO provides a small contribution to the external quantum efficiency as discussed above and for this reason the term NiO/FeMnO_3_-based heterojunction solar cells is used within the paper. The integrated photocurrent density (0.064 mA/cm^2^) is also in close accord with the value obtained from the J–V curve (0.07 mA/cm^2^) acquired from the solar simulator analysis.

EIS measurements were performed to obtain further insights into the depletion regions of the p−n device under study and to further understand the low generated photocurrent. Previous reported EIS measurements on lead free perovskite oxides have been performed at Voc conditions (Sariful Sheikh et al., 2017). Our trials to measure at Voc conditions resulted to low signal and high noise from moderate to high frequencies that do not enabled the analysis of EIS parameters. Thus, the presented measurements were performed under illumination and at Jsc conditions which provided adequate signal for the analysis of the EIS measurements. Figure 5C, shows characteristic Nyquist plots of the NiO/FeMnO_3_ heterojunction structure as well as the equivalent circuit model used to fit the experimental data; Even though the model does not perfectly much the experimental results is used to provide initial analysis of the EIS measurements presented. Specifically, the model is commonly used with the components R_1_, R_3_ and R_3_ being ascribed to the contacting, charge transport and recombination resistance, respectively (von Hauff, 2019). The obtained results shown in Table 2 indicate that R_3_ > R_2_ by an order of magnitude and so we can infer that the ITO/NiO/FeMnO_3_/Cu device exhibits a relative high recombination resistance which can explain the high V_oc_ value, but on the other hand shows a high charge transport resistance which results in limited current density. We note that the value of recombination resistance is expected to be lower at Voc conditions due to the absence of the depletion’s layer driving force formed by the applied Jsc measuring conditions. In Figure 5D, the Mott-Schottky measurements of the title device which was swept from low to high external applied bias are illustrated. According to Mott-Schottky analysis the crossing of extrapolated linear section of the spectra with *x*-axis can be ascribed to the built-in potential of the device. Assuming that the proposed device is fully depleted during the measurement we exact a built-in potential of 1.38 V (von Hauff, 2019).

**TABLE 2:** Parameters obtained by fitting the Nyquist plots of the ITO/NiO/FeMnO_3_/Cu device.

Solar cell	R_1_ (KΩ)	R_2_ (MΩ)	C_1_ (nF)	R_3_ (MΩ)	C_2_ (nF)
ITO/NiO/FMO/Cu	1.83	5.52	0.053	57.27	0.059

Overall, to achieve higher PCE from the presented solution processed NiO/FeMnO_3_ heterojunction inorganic photovoltaics, further research and material development methods are needed. The main limiting factor that must be addressed is the improvement of n-type FeMnO_3_ charge transport properties and thickness optimization of the active layer to decrease the charge carrier recombination. In parallel, incorporation of buffer layers within the ITO/NiO/FeMnO_3_/Cu device structure can be used to improved charge carrier selectivity as well as a more appropriate p-type material can be applied with higher light harvesting capabilities and better aligned VB level edge to n-type FeMnO_3_ to facilitate the holes transfer. The above proposed research and material development efforts can result to higher PCE for solution processed based heterojunction inorganic photovoltaics.

## Conclusion

This study successfully proves that the synthesis of FeMnO_3_ NPs can be achieved by a solution combustion technique, using tartaric acid as a fuel. Furthermore, the ultimate control of the nanoparticle’s size can be readily attained due to the multiple binding ability of the tartaric acid that resulted in the formation of single-phase FeMnO_3_ with an average particle size of 13 ± 2 nm. X-ray diffraction and electron microscopy measurements verified the high phase purity and crystallinity of FeMnO_3_. Additionally, we used a method to construct a network of tightly connected FeMnO_3_ nanoparticles by spin coating of the colloidal solution. In this study, *β*-alanine was used as surface capping agent to produce a stable colloidal dispersion of FeMnO_3_ NPs (*β*-alanine-capped FeMnO_3_ NPs) in DMF. The short chain length of *β*-alanine allows direct interactions between the nanoparticles through ligand removal by thermal annealing (at 450°C in air), thus yielding a thick absorbing film (∼500 nm) consisting of continuous layers of interconnected nanoparticles and exhibiting a relative low roughness of ∼27 nm. The proposed strategy is crucial to obtain functional all-oxide photovoltaic devices that are developed using a process technique based on a simple solution. Furthermore, the inorganic perovskite FeMnO_3_ was tested as a light absorber for photovoltaic applications for the first time. The band gap (∼1.54 eV) of the synthesized FeMnO_3_ nanostructure was found to be very close to the hybrid lead perovskite CH_3_NH_3_PbI_3_ material (∼1.55 eV). To this end, all-inorganic NiO/FeMnO_3_ heterojunction photovoltaics were fabricated by solution combustion synthesis, using spin coating techniques. The corresponding all-inorganic solar cells reveal a high open circuit voltage (V_oc_) of 1.31 V with a fill factor (FF) of 54.3% but exhibit a PCE of 0.05% under 100 mW cm^−2^ illumination due to the limited short circuit current 0.07 mA cm^−2^. Electrical characterization by impedance spectroscopy showed that the ITO/NiO/FeMnO_3_/Cu device exhibits a high recombination resistance justifying the high V_oc_. The high charge transport resistance indicates charge transport limitations within the relative thick (∼500 nm) n-type FeMnO_3_ active layer. Optimizing the active layer thickness and improving the charge carrier transport properties are the main limiting processes of the n-type FeMnO_3_ which results in low J_sc_ values for the presented solution processed based heterojunction inorganic photovoltaics. Moreover, further PCE improvement could be achieved by the incorporation of suitable charge selective contacts within the solar cell device architecture and the replacement of NiO with a more appropriate p-type material. The obtained results encourage more intense research on solution processed and environmentally friendly inorganic solar cells with suitable opto-electronic properties and high photon to electron conversion efficiency.

## Data Availability

The raw data supporting the conclusion of this article will be made available by the authors, without undue reservation.
