# Supplementary material for: All-Inorganic p−n Heterojunction Solar Cells by Solution Combustion Synthesis Using N-type FeMnO3 Perovskite Photoactive Layer
Source: Front Chem. 2021 Sep 29;9:754487. doi: 10.3389/fchem.2021.754487 (PMC8511641; doi:10.3389/fchem.2021.754487)
Supplement: Supplementary file 1 [file DataSheet1.pdf]

Supporting information

All-Inorganic p−n Heterojunction Solar Cells by Solution Combustion Synthesis using n-type $FeMnO_3$ Perovskite Photoactive Layer

Ioannis T. Papadas,[a,c]* Apostolos Ioakeimidis,[a] Ioannis Vamvasakis,[b] Polyvios Eleftheriou [a] Gerasimos S. Armatas[b] and Stelios A. Choulis[a]*

[a] Molecular Electronics and Photonics Research Unit, Department of Mechanical Engineering and Materials Science and Engineering, Cyprus University of Technology, Limassol, Cyprus.

[b] Department of Materials Science and Technology, University of Crete, Heraklion 70013, Greece.

[c] Department of Public and Community Health, School of Public Health, University of West Attica, Athens, Greece

*** Correspondence:**

Corresponding Authors: Assistant Prof. Ioannis T. Papadas, Prof. Stelios A. Choulis

emails: ioannis.papadas@cut.ac.cy, stelios.choulis@cut.ac.cy

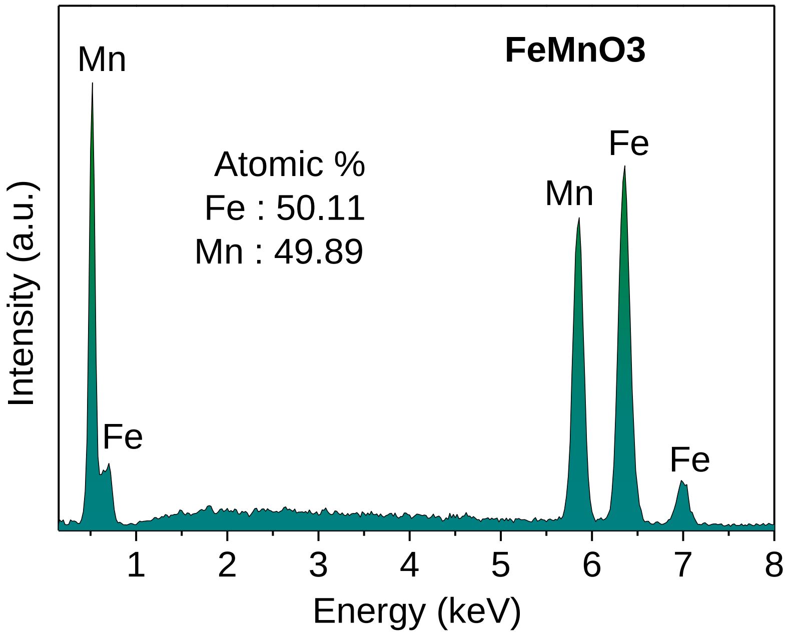

Fig. S1. Typical EDS spectrum for $FeMnO_3$ nanoparticles. The EDS analysis indicates an average atomic proportion of Fe:Mn ~1:1.

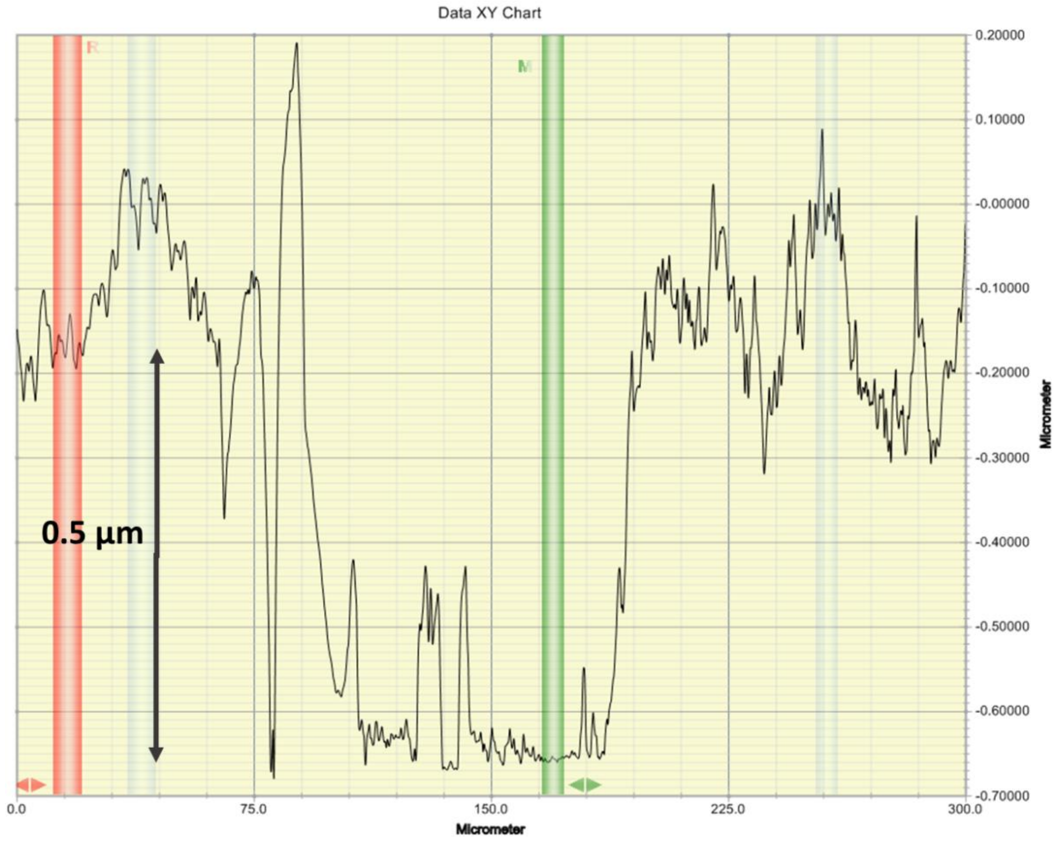

Fig. S2. Surface profile measurements of $FeMnO_3$ nanoparticles thick layer (~500 nm) under study.

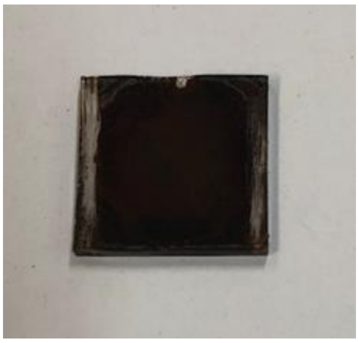

Fig. S3. Photograph of $FeMnO_3$ nanoparticles film obtained by spin coating technique.
